# A treadmill training program in a gamified virtual reality environment combined with transcranial direct current stimulation in Parkinson’s Disease: Study protocol for a randomized controlled trial

**DOI:** 10.1371/journal.pone.0307304

**Published:** 2024-07-16

**Authors:** Pere Bosch-Barceló, Carolina Climent-Sanz, Oriol Martínez-Navarro, Maria Masbernat-Almenara, Anni Pakarinen, Pradip K. Ghosh, Helena Fernández-Lago

**Affiliations:** 1 Department of Nursing and Physiotherapy, University of Lleida, Lleida, Spain; 2 Grup de Recerca de Cures en Salut, Lleida Institute for Biomedical Research Dr. Pifarré Foundation (IRBLleida), Lleida, Spain; 3 Lleida Institute for Biomedical Research Dr Pifarré Foundation, IRBLleida, Lleida, Spain; 4 Society, Health, Education and Culture Research Group (GESEC), University of Lleida, Lleida, Spain; 5 Department of Nursing Science, University of Turku, Turku, Finland; 6 Physical Therapy Program, Walker College of Health Profession, Maryville University, St Louis, MO, United States of America; Public Library of Science, UNITED STATES OF AMERICA

## Abstract

**Background:**

Parkinson’s Disease (PD) affects movement and cognition, and physiotherapy, particularly treadmill gait training, has potential in addressing movement dysfunctions in PD. However, treadmill training falls short in addressing cognitive aspects and adherence. Virtual reality (VR) and gamification can enhance motor and cognitive retraining and improve adherence. People with Parkinson’s Disease (PWPD) have decreased motor skill learning efficiency, but tDCS can improve motor and cognitive learning.

**Methods:**

78 participants with PD will be randomly allocated in a 1:1:1 ratio to one of three groups: (1) treadmill + Gamified Virtual Reality Environment (GVRE) + tDCS training group; (2) treadmill + GVRE training group or (3) treadmill training group. Participants will follow a 6-week, 12-session treadmill gait training plan, gradually increasing session duration from 20 to 45 minutes. Participants in (1) and (2) will undergo a GVRE training protocol, with (1) also receiving tDCS for the first 20 minutes of each session. Assessments will occur at baseline, post-intervention, and at a 6-week follow-up. The primary outcome measure will be gait speed during single and dual-task performance. Secondary measures will include additional gait parameters, executive tests for cognitive performance, and clinical outcomes for disease stage, cognitive status, and physical condition.

**Discussion:**

This randomized clinical trial presents an innovative neurorehabilitation protocol that aims to improve gait and cognition in PWPD. The study also examines how tDCS can enhance motor and cognitive training. Results could contribute to enhancing the motor and cognitive state of PWPD through a GVRE and tDCS-based neurorehabilitation protocol.

**Trial registration:**

NCT05243394. 28/02/2024 –v3.2

## 1.- Introduction

Parkinson’s Disease (PD) is a neurodegenerative disorder mainly known to cause resting tremor, bradykinesia, postural instability, rigidity, and a highly variable, short-stepped gait [1–3]. People with Parkinson’s Disease (PWPD) also have a wide variety of non-motor difficulties that affect their daily activities. It has been recently shown that they present a slight cognitive decline at the early stages of the disease that aggravates with time [4]. This could explain why there is an increase in falls and freezing of gait while carrying out several tasks at the same time [5]. This combination of gait and motor or cognitive tasks is known as Dual Task (DT), and is related to gait impairments such as reduced speed and stride length, decreased symmetry and coordination and increased variability [6].

Since PD is a chronic disease that has no cure, pharmacological treatment with Levodopa is the mainstay for management of motor symptoms. However, as the disease progresses most patients need increasing doses and end up with side effects including dyskinesia and on-off fluctuations [7]. Levodopa improves hypokinetic gait in the early stages of the disease [8]. However, levodopa responsiveness on gait worsens with time, and some parameters, such as gait timing, are not affected by levodopa [8]. Moreover, the presence of non-motor symptoms such as cognitive impairment, depression or psychosis creates even more treatment challenges. In this context, physiotherapy has shown promising results as an effective tool in rehabilitating gait dysfunctions in PWPD through therapeutic exercise [9]. One of the most used therapies consists in walking on a treadmill several times a week, showing positive effects over physical performance in PD, improving walking economy and the specific gait kinematic parameters affected in PD: speed, stride length and gait variability [10–13]. The feasibility and safety of treadmill gait training at moderate and high intensities have both been well established in de novo PD [14].

However, this type of intervention fails to address the importance of cognition for safe walking [15]. PWPD also have difficulties in following exercise programs for extended periods of time due to fear of falling, lack of expectations, apathy, and fatigue [16]. Virtual Reality is a computer-generated technology that allows the user to interact with a virtual environment similarly to a physical place. In the Virtual Reality environment, PWPD have access to amplified feedback on their performance and are able to train both motor and cognitive abilities. This environment offers the chance to learn and retrain motor and cognitive strategies that may have been lost due to PD’s degenerative nature [17–19]. Current evidence presents Virtual Reality as a promising tool for PWPD that could optimize motor learning by offering a safe and realistic environment in which individualized and repeated motor function can be practiced while stimulating cognitive processes at the same time [19].

Gamification, which is generally defined as “the use of game design elements in non-game contexts” [20] seeks to recreate an environment to enhance experiences and motivation. Based on Ryan and Deci’s Self-Determination theory [21], participants would be driven by their need to grow and gain fulfillment, making gamification a more helpful tool in turning exercise into a more engaging activity, which could improve adherence [22, 23]. More specifically, gamification could be a beneficial tool for physiological, mental and cognitive functions, even though available literature on elderly populations is scarce so far [24].

A relevant factor in the rehabilitation of gait in PWPD is the efficiency with which motor skills are learned. In comparison with healthy individuals, PWPD show a reduced consolidation and automatization in motor skill learning, which they attempt to compensate for by recruiting additional brain regions and by altering the effective connectivity patterns [25]. Through practice patients are able to improve their performance, though their ability to learn new motor skills and consolidate the achieved skills remains affected [25]. In this sense, Transcranial Direct Current Stimulation (tDCS) is presented as one of the techniques of choice to improve the learning of motor skills in PWPD. However, this technique is currently a topic of intense scientific discussion due to the conflicting results from previous studies [25].

A systematic review by *Broeder et al*. [25] concluded that tDCS can have significant improvements on motor and cognitive functioning in patients with PD. Specifically, anodal-tDCS, which increases cortical excitability, showed positive effects on several parameters of gait performance in 5 out of 7 different studies, with one of them finding significant benefits of combining anodal-tdcs with physical training. tDCS has the capacity to enhance or suppress widespread neuronal activity, leading to the release of dopamine via motor networks within the basal ganglia-cortex-cerebellum system and other motor cortical regions [26]. According to research by *Frase et al*. [27], repeated anodal-tDCS sessions would be able to directly trigger plastic Long-Term-Potentiation-like effects in the human cortex.

Effects of tDCS on the improvement of motor functions may be caused by the activation of motor and prefrontal cortex regions, which may lead to the release of dopamine in PWPD, thus promoting the improvement of motor functions [28]. Current evidence also seems to point towards certain benefits of anodal-tDCS when combined with therapeutic exercise, such as an improvement of cognitive functions [29]. However, the diverse variations in stimulation parameters and target regions across different studies prevent definitive conclusions [28]. Within the realm of tDCS, various approaches have been suggested. Bi-hemispheric stimulation, while potentially cost-effective for concurrent cognitive tasks, lacks conclusive evidence at present [30, 31]. Even within this scarce body of evidence, no studies have provided insight on how its use in combination with a Gamified Virtual Reality Environment (GVRE) may influence motor and cognitive learning. Therefore, diving deeper in the short- and mid-term effects of gait training in a GVRE would help to delineate whether tDCS could provide any additional benefits in PWPD.

The aim of this three-arm RCT is to evaluate the effects of the inclusion of a GVRE to a 6-week treadmill training program with anodal-tDCS in PWPD on gait parameters during Single and Dual Task, clinical outcomes and executive functions; in comparison to a GVRE treadmill training program; and compared to a treadmill training as a control.

## 2.- Materials and methods

### 2.1.- Trial design

This is a prospective, single-blinded, single center, three-arm RCT. This study protocol follows the Standard Protocol Items: Recommendations for Interventional Trials (SPIRIT) guideline for clinical trial protocols.

**Fig 1:**
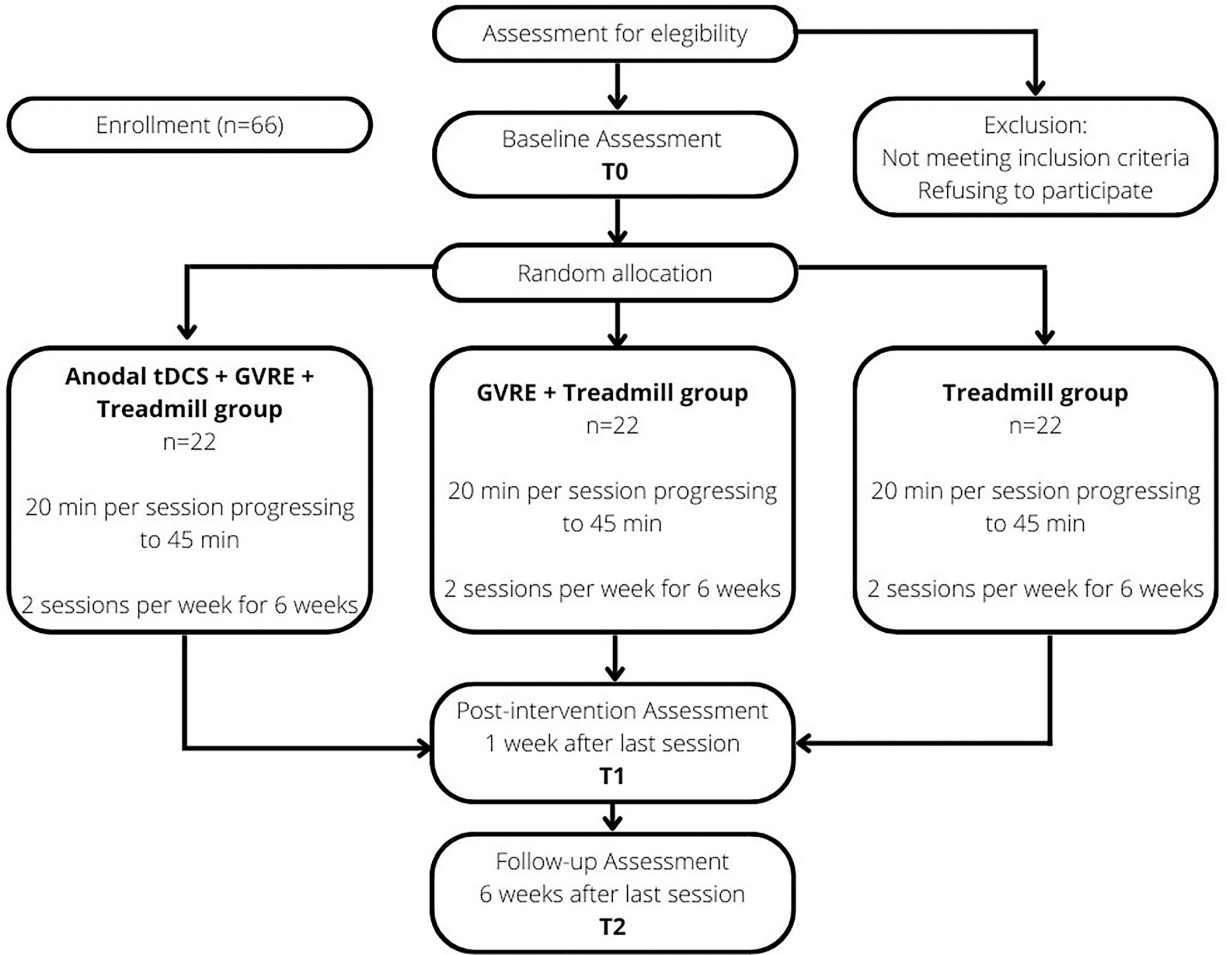
Flow diagram based on CONSORT.

The study will be conducted in Lleida, Spain, at the Lleida Institute for Biomedical Research Dr. Pifarré Foundation. Eligible participants will be randomly distributed in three groups: 1) treadmill gait training; 2) GVRE + treadmill gait training; 3) anodal-tDCS + GVRE+ treadmill gait training. Fig 1 shows a flow diagram based on the Consolidated Standards of Reporting Trials (CONSORT) for this study. Table 1 shows the planned schedule to address enrollment, assessments (at baseline, post-intervention and follow-up) and interventions.

**Table 1:** Schedule of enrollment, interventions and assessments.

	Study Period			
	Enrollment	Allocation	Post-allocation	Follow-up
Timepoint	-t_1_	t_0_	t_1_	t_2_
**Enrollment**				
Eligibility Screen	x			
Written Informed Consent	x			
Allocation		x		
**Interventions**				
Intervention G1			x	
Intervention G2			x	
Intervention G3			x	
**Assessments**				
Executive function assessment (Victoria Stroop test, Go/No-Go test)		x	x	x
Clinical outcomes (MoCA, UPDRS, H&Y, PDQ39, MiniBEST, FES-I)		x	x	x
Gait Assessment under Single and Dual Task conditions		x	x	x

Baseline assessments (T0) will provide information regarding motor, cognitive and clinical data before the intervention takes place. In the next session after the intervention is over, a post-intervention assessment (T1) will be carried out to know the effects of the training protocol as well as how both groups’ results compare. Finally, after 6 weeks post-training (T2), a follow-up assessment will be performed to measure how any results the participants may get through the intervention retain over time. All participants will perform their interventions at the same hour of the day and during the ON phase of medication to avoid any bias related to circadian rhythms or ON-OFF fluctuations. This measure will be encouraged by asking participants to take their medications 1 hour previous to meeting the researchers to be evaluated. All evaluations will follow the same timing restrictions. A researcher blinded to the participants’ allocation will conduct all assessments.

All procedures have been approved by the ethical committee of Hospital Universitari Arnau de Vilanova (CEIC-2231), and both the Helsinki Declaration and the Oviedo Convention will be followed [32, 33]. Spanish regulations regarding Biomedical Research will be met as well [34].

### 2.2.- Sample size

The sample size calculation was based on the study of Mirelman et al [35]. From this study, we assumed that gait speed during a treadmill dual-task assessment in PD patients would be approximately 1.01±0.23 m/s. From the study of *Hass et al*. [36] it was established that an increase of 0.22 m/s in gait speed would be considered clinically relevant. The GRANMO sample size calculator [37] was used to run an analysis of variance calculation.

With an alpha risk of 0.05 and a beta risk of 0.20, assuming a 10% withdrawal rate, and using a two-sided test, it will be necessary to include 26 subjects in each of the three groups.

### 2.3.- Participants

In total, 78 participants will be recruited mainly via the Hospital Universitari Arnau de Vilanova of the city of Lleida, as well as through contact with neurologists, local PD associations (Associació de Parkinson de les Terres de Lleida) and other methods such as distribution of fliers and contact with local news media.

The following inclusion criteria will be applied:

- Aged 50 or older
- Idiopathic PD (UKPDSBBC)
- Stage II–III in the Hoehn & Yahr (H&Y) scale during ON state
- Ability to walk for 10 minutes independently without stop

The exclusion criteria will be the following:

- Significant cognitive decline based on mini mental status examination (MMSE <23)
- Severe auditory or visual deficits
- Other neurological/psychiatric conditions
- Any kind of cardiovascular complications that contraindicates physical activity
- Clinical history of any brain surgery or deep brain stimulation device

Subjects that must change their medication while participating in the RCT will be considered as non-retention subjects.

### 2.4.- Ethics approval

This study was authorized by the Comité d’Ètica d’Investigació amb Medicaments (CEIM)–Hospital Universitari Arnau de Vilanova, Lleida, Spain, with identifier CEIC-2231.

Any protocol amendments or changes will be notified to the ethics committee as well as updating all information in the clinical registry.

Participants will give their individual, signed written informed consent to participate in the study and will be thoroughly informed about all procedures that will be carried out by an evaluator. Adherence will be promoted with session reminders as well as progression tracking.

### 2.5.- Randomization and blinding

Randomization and allocation concealment will be performed using a random number generation computer technique. Randomization will be made by blocks with a size of 3 and stratified based on two covariables: age and disease stage. The ratio of assignment will be 1:1:1, and the randomized list will be generated through the Study Randomizer software by a blinded postdoctoral researcher who will not be involved in the study. Opaque, sealed envelopes will be used to conceal the allocation sequence, and opened only at the time of randomization in the presence of the study participants.

### 2.6.- Interventions

The intervention program will last for 6 weeks with 2 sessions/week for a total of 12 sessions. This general training protocol structure is based on studies from *Mirelman et al*. [15, 38] and *Shulman et al*. [39], and adapted to fit the GVRE and training characteristics. A 2-session per week instead of 3 will be implemented for two reasons: there seems to be no evidence pointing to better results in interventions having 2 or 3 sessions per week, and the dispersion of elderly population in Lleida will make it easier for participants to attend training with a slightly lower frequency [19, 40]. All changes to the original training protocol structure are based on feedback from a feasibility study performed before the RCT with PWPD and physiotherapists [41]. First-hand information was collected regarding difficulties found and improvements that could be made while using the GVRE rehabilitation system. Improvements were made in fixing of issues within the software and adjusting the training duration, frequency and speed increases.

During the sessions, all participants will be secured by a harness with no weight liberation attached to a safety structure to ensure there is no risk of falls. All sessions will be supervised by at least one physiotherapist with experience managing PWPD, neurorehabilitation and tDCS.

Exercise intensity will be monitored based on the heart rate reserve (HRR; the difference between resting heart rate and maximum heart rate) [42]. Perceived exertion will be recorded using the Borg scale [43]. Moderate intensity (60% of HRR or up to 6 on the Borg scale) will not be exceeded in the sessions. In any case where participants exceed safe intensity levels, any block in progress will be stopped and the rest period will start immediately. Any issues will also be communicated to the participants’ neurologist.

#### Group 1: Treadmill + GVRE + tDCS

**Fig 2:**
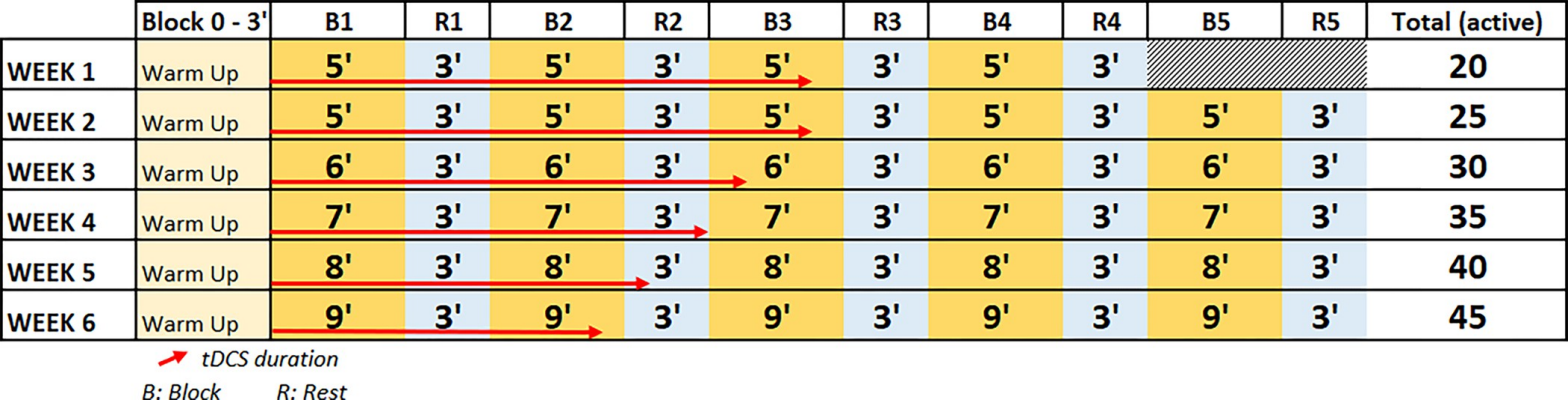
Progression of training duration and tDCS application.

This group will follow a treadmill gait training protocol that will last for a total of 6 weeks. Each participant will attend 2 sessions every week, for a total of 12 sessions. All sessions will begin with a 3-minute warm up block walking on the treadmill with no GVRE. The duration of sessions will increase as weeks progress, from a starting duration of 20 minutes to a final duration of 45 minutes. The first week will comprise 4 blocks of 5 minutes each. On the second week, one block will be added, for a total of 5 blocks of 5 minutes. Weeks 3 to 6 will also have 5 blocks of increasing duration, from 6 to 9 minutes, respectively. A 3-minute rest will be given after each block on all weeks (Fig 2).

The treadmill speed for the first week will be set at 85% of the normal walking speed achieved at the 10-meter walking test on the ground. The second week will be at 90% of that speed. Weeks 3 to 6 will all have a set speed based on a 5% increase on the previous week’s speed.

**Fig 3:**
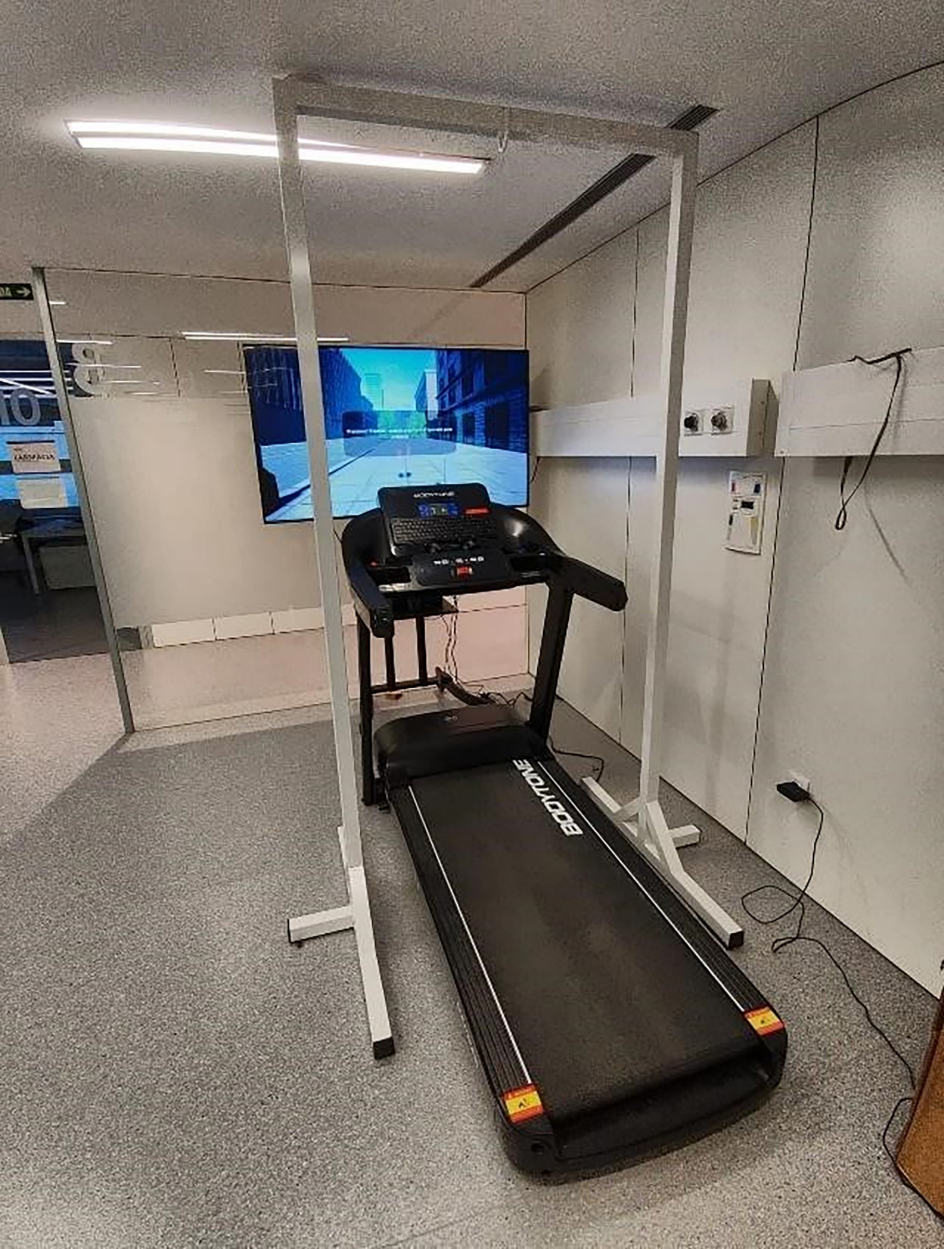
GVRE + treadmill set-up.

Along with the treadmill gait training, a GVRE will be applied to this group by setting a wide screen TV in front of the treadmill. Two HTC SteamVR 2.0 sensors will collect information from two HTC Vive 3.0 Trackers strapped to the participants’ feet. This data will be processed by the computer and represented in the GVRE on the screen [44] (Fig 3).

**Fig 4:**
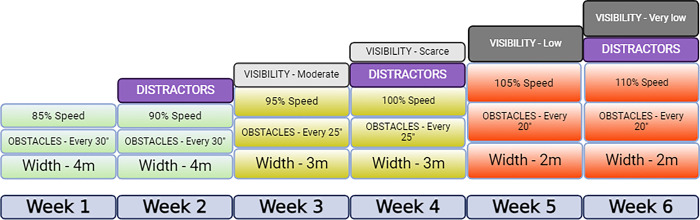
Increase of the difficulty through the modification of mentioned variables.

This way, the participant will be able to interact with the virtual environment by altering his walking patterns on the treadmill. The GVRE involves the participant in a “walking your dog” task, allowing the participant to choose among different colors for the dog and naming it. The dog will walk besides the participant for the entirety of every session [23]. The dog animation will change along the training, with a slow walking animation on the first two weeks, a faster walk on the third and fourth, and a light trot on the fifth and sixth weeks, so that all participants experience the feeling of improvement of the dog increasing its speed. The other gamification elements include a progress bar, green/yellow/red indicators depending on performance, difficulty progression, customized leveling up and score systems. Three different virtual environments will be created: park, city and countryside, and the training will be carried out for 2 weeks on each one of them. Level of difficulty in the GVRE will be modified through 5 different mechanisms: speed (as explained previously), obstacles, distractors, visibility, and path width (Fig 4).

**Fig 5:**
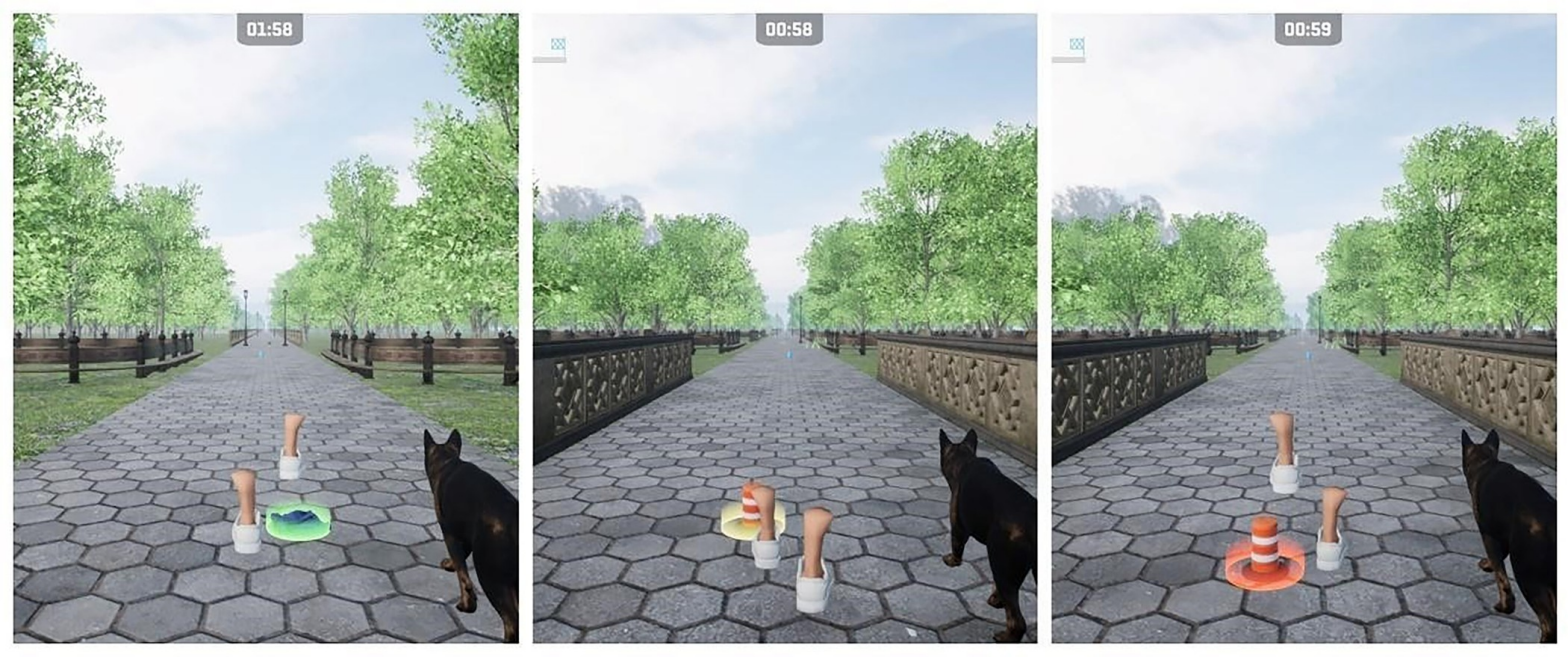
Gamification feedback on obstacle management.

Obstacles will depend on the environment initially set, but will include bricks, traffic cones and other similar items. During the first and second weeks, obstacles will appear every 30 sec., forcing the participant to dodge them by modifying their walking pattern on the treadmill. For weeks 3 and 4 obstacles will appear every 25 sec., and it will be reduced to every 20 sec. on weeks 5 and 6. Whenever the participant gets close enough to an obstacle, the object will light up with an orange circle to encourage a dodging step, and it will turn green or red depending on whether there was a collision or not (Fig 5). This will work as feedback for participants to better calculate distances during the rest of the session.

Distractors will appear on weeks 2, 4 and 6, and will comprise different sounds and visual information to alter the participant’s focus. Examples of this will be crossing animals making sounds, rivers, building sites and people walking by, depending on the environment.

**Fig 6:**
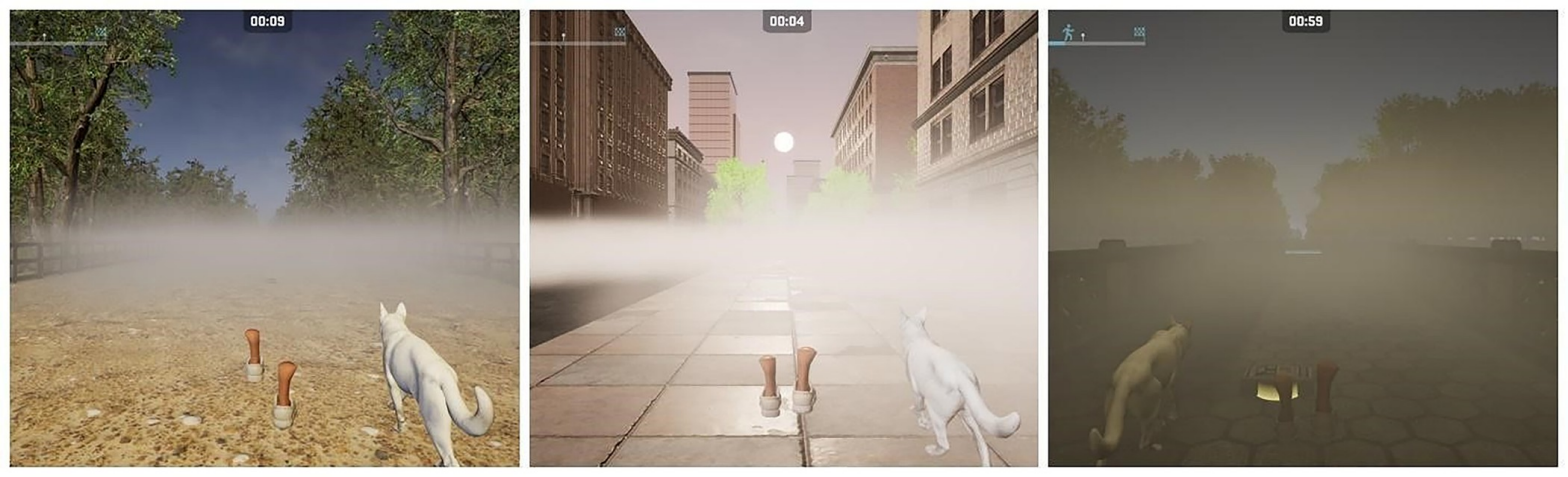
Difficulty increase based on visibility limitations.

Visibility will be altered in two ways: by changing the environmental light and via the introduction of fog. The first week will be carried out in morning daylight and no fog; the second week will be at noon with no fog; the third week will be in the morning and will have light fog at the latter half of the blocks; the fourth week will be during evening and will have medium fog at the two final blocks; the fifth week will be during evening and will have medium fog at the two final blocks, and the sixth week will be during nighttime and thick fog will be used at the two final blocks (Fig 6).

Path width will be altered to challenge the participants to walk in a more saturated environment: weeks 1 to 2 will comprise a 4-meter pathway, which will be reduced to 3 meters in weeks 3 and 4 and down to 2 meters in weeks 5 and 6.

Participants will also be able to go up one level after the first session if they complete 3 blocks with over an 80% success in obstacle management. This will be limited to only one level promotion per week. Visual metrics will be used to reinforce the feeling of reward when successfully finishing blocks. Moreover, leaderboards regarding scores on obstacle management will be shown to participants in order to keep them engaged and motivated throughout the training.

This group will also receive the treadmill gait training protocol on the virtual reality environment with an anodal-tDCS intervention. Each session will begin with the anodal-tDCS setting. This will consist in placing two turned off sponge electrodes with a total surface of 35cm^2^ on the participant’s scalp after applying a saline solution. They will be set in place using straps. The anode will be placed over the left-Dorsolateral Prefrontal Cortex (DLPFC), identified as the F3 position based on the 10–20 International EEG System, and the cathode will be placed over the right DLPFC [30, 31]. After the 3-minute warm-up block, the anodal-tDCS will be turned on. The anodal-tDCS stimulation will last for 20 minutes, with an intensity of 2mA, which will be reached after a 30-second wind-up, and it will not be stopped during walking breaks (Fig 2). This stimulation protocol using anodal-tDCS has shown improvements in dual-task gait performance compared to sham stimulation in previous research [45, 46].

#### Group 2: Treadmill + GVRE

Group 2 will follow the same treadmill training protocol as group 1, but will have no anodal-tDCS stimulation.

#### Group 3: Treadmill group

This group will follow the previously explained treadmill gait training protocol with no virtual reality environment nor anodal-tDCS intervention.

### 2.7.- Outcome measures

The main outcome measure will be speed, measured in both the Intermittent Shuttle Walking Test and during Dual Tasking. The rest of gait parameters will be accounted as secondary outcome measures:

Gait parameters during the Intermittent Shuttle Walking Test: Participants will walk along an 8-meter-long zone for 10 round trips at 5 different speeds: preferred, slow, very slow, fast, and very fast (10x5x8m). Based on *Ambrus et al*. [47] this protocol will allow for a lineal relation between stride length and speed to be established with high confidence. During the walk, the following cinematic gait parameters will be recorded via an inertial sensor system APDM® (Opal, APDM, Portland, OR, USA): speed (m/s), stride length (m), gait cycle cadence (Hz), stride length variance coefficient and variance coefficient of the gait cycle cadence.

Gait parameters during Dual Task: Participants will walk along the same aisle as described in the previous variable while carrying out a simultaneous task. Three different types of Dual Task will be assessed: **Mental tracking:** The Mental Tracking Dual Task consists of walking while subtracting 7 serially from 100; **Verbal Fluency**: The Verbal Fluency Dual Task consists of walking while naming words beginning with a specific letter; **Motor Dual Task**: The Motor Dual Task consists of walking while carrying a tray with both hands that has a half-filled glass of water on it. Performance on Mental Tracking Dual Task and Verbal Fluency Dual Task will be assessed in order to evaluate attentional processes during gait [48]. Participants will not be instructed to prioritize the gait nor the DT during the assessment.

To avoid practice or fatigue effects, the Dual Task order will be randomized. Each task will be measured 3 times, resting 1 minute between each try, and using mean data for analysis.

The secondary outcomes and descriptors will include:

Executive function assessment:

In order to assess the participants’ executive functions, two different executive function assessments will be taken via PEBL2 (The Psychology Experiment Building Language), a free software available from http://pebl.sf.net. These executive function tests will measure the response inhibition, reaction time, attention and management of interferences of participants; all related to the GVRE experience. These tests will be the following:

- Victoria Stroop Test, which will measure the ability to inhibit cognitive interference [49]. A total of 3 blocks with different words and color stimuli will be assessed, with time taken, responses made and interference as measuring units.
- Go/No-Go Test, which will measure the participant’s attention and response control [50]. A total of 80 stimuli will be provided, with 20% of those being fake signals that must be ignored. A total accuracy score based on participant responses will be calculated.

Clinical outcomes:

The following scales and tests will be used to assess clinical symptomatology as well as aspects related to quality of life and daily activity autonomy.

Unified Parkinson’s Disease Rating Scale–Part III (UPDRS): UPDRS will be used to assess and monitor PD related disability and impairments. Part 3 of this scale comprises an assessment of different aspects of motor function [51].

Hoehn & Yahr: The assessment using H&Y scale is based on issues of unilateral versus bilateral involvement and the presence or absence of postural reflex impairment [52].

Parkinson’s Disease Questionnaire 39 (PDQ39): This is a patient reported 39-item questionnaire and it assesses PD-specific health related quality of life and disease specific health status [53].

MiniBest Test: This 14-item test is used to assess dynamic balance based on anticipatory postural adjustments, reactive postural control, sensory orientation and dynamic gait [54].

Falls Efficacy Scale International (FES-I): FES-I is a 16-item self-administered questionnaire including a range of functional activities and is designed to assess fear of falling [55].

Montreal Cognitive Assessment (MoCA): MoCA is a 30-question test to assess cognitive function and it evaluates orientation, short-term memory, executive function/visuospatial ability, language abilities, abstraction, animal naming, and clock-drawing test [56].

Any information regarding all the above measurements can be found in http://www.scalesandmeasures.net/. Moreover, a semi structured interview will be added in regards to adherence to treatment, which will allow for a qualitative analysis to explore the experience regarding the GVRE.

Participants will be reminded not to inform the evaluator about their group assignment in order to ensure blinding.

Potential adverse events may include headaches, dizziness and discomfort, due to the GVRE, and skin irritation or redness at the site of tDCS electrode placement during or after the intervention. There may also be issues with training intensity in some cases. However, these risks are considered low and can be minimized through proper screening of participants, monitoring during the intervention, and adequate follow-up.

### 2.8.- Data collection, management, and dissemination

Demographic data such as age, sex, height, disease duration and most affected side will be collected. Data from outcome measures will be collected at T0, T1 and T2.

In order to maintain confidentiality, all participants will be assigned an identification number through randomization and all data from each participant will be recorded only using the assigned number for each participant. All measurements will be recorded on data sheets. Once the data sheet has been copied to a document in a password protected digital file, the data sheet will be shredded. Other documents to be stored in a locked cabinet include the master list linking the participant’s name to their number as well as contact correspondence. A database validation will be carried out considering the variable range and missing values.

The final trial results will be written into scientific papers and sent to peer reviewed, high quartile journals to disseminate knowledge among the scientific community.

### 2.9.- Statistical analysis

Statistical analysis will be conducted on a blinded intention-to-treat approach. Alfa error will be set at 0.05. The descriptive statistics will use frequencies for the categorical variables and measures of central tendency such as the mean and standard deviation, median and interquartile range for the quantitative variables. Sociodemographic baseline characteristics will be assessed between groups through descriptive statistics. The normality of data distribution and homogeneity of variances will be determined by using the Kolmogorov-Smirnov and Levene’s tests, respectively. The presence of Freezing of Gait will be taken into account as a confounding factor during the analysis process.

Using a mixed methods regression model, data for each variable will be compared between subjects’ factor (Treadmill group, Treadmill + GVRE group, Treadmill + GVRE + anodal-tDCS) and within subjects’ factor on the time points (baseline, post-treatment and a follow-up after 6 weeks of last intervention). Any non-normally distributed data will be log transformed [57]. Multiple imputation by chained equation will be used to manage missing data [58].

Results will be presented as estimated differences with 95% confidence intervals. Gait speed changes will also be expressed in terms of clinically meaningful difference. Based on the study by Hass et al. [36] clinically meaningful differences will be small if the difference is 0.06 m/s, will be moderate if the difference is 0.14 m/s, and will be large if the difference is 0.22 m/s.

## 3.- Discussion

PD is the fastest growing neurodegenerative disorder, and the progressive aging of the population will result in an even larger increase in prevalence of neurodegenerative diseases and age-related conditions [59]. PD can greatly hinder an individual’s ability to perform motor skills such as walking, turning around as well as transferring in and out of bed [60]. Gait disorders are major contributors of functional disability in individuals with PD and drastically impact quality of life [61]. Difficulties in walking with altered movement patterns along with abnormal posture are important contributors to an increased fall risk [62].

Most symptoms are initially managed by medications by providing a pharmacological substitution of dopamine to the brain. However, efficacy of these medications reduces over time. Moreover, medications cannot fully address and control motor symptoms throughout an individual’s lifetime. Current evidence seems to point at exercise interventions as the best course of action for improving movement and gait in individuals with PD and reducing their fall risk [62]. A wide range of exercise interventions are used with the objective of improving gait in PD, but currently there are no recommendations on superior interventions [63]. Despite this large variety of available exercise interventions, little research has been done to show the effects of gamification in combination with virtual interventions. Gait training on a treadmill in gamified virtual environments could be a novel approach and will be a cheap, new, and motivating rehabilitation modality. Because of its ability to optimize motor feedback through increased feedback on performance, and repetitive training on motor functions along with simultaneous stimulation of cognitive processes, gait training in a virtual environment would be an effective choice to improve gait and even cognitive functions. However, mechanisms through which patients improve their functional capacity and cognition during exercise training are still unknown. The combination of physical activity and non-invasive brain stimulation with anodal-tDCS could improve the learning process in these patients. All of this sets this intervention as a potential strategy in rehabilitation of patients with PD. Moreover, the RCT proposed in this paper stands out in its unique user-centered design, putting the end-user at the middle of the equation by testing the rehabilitation system’s usability and feasibility before carrying out the RCT.

In this proposed study, gait parameters will be assessed both during walking and as well as walking with DT via the Shuttle test. This will allow for a relation between stride length and speed to be established with high confidence based on studies from *Ambrus et al*. [47]. Assessment of secondary variables including balance, daily activities as well as quality of life will provide further insights about the influence of a virtual environment during walking on other functional activities as well as health related quality of life. Experimental set-ups in three different groups will allow us to differentiate between the effects provided by every layer added to the base treadmill training. Through this study design, we will also be able to quantify how participants react to a treadmill-based gait training protocol, how the inclusion of a GVRE can possibly further enhance the improvements and whether anodal-tDCS is effective in lengthening the duration of the beneficial effects both in the short and the long term. The progressive manner of the training protocol will allow us to study how patients react to each different training environment and the adaptations that they achieve to overcome the progressive difficulty in both physical and *psychological* domains.

In summary, this trial will compare the effects of treadmill gait training in a GVRE and with anodal-tDCS on gait performance and cognitive aspects in patients with PD. The results obtained will be useful to clarify how virtual reality and anodal-tDCS can help patients with PD improve their physical and mental condition and retain the benefits for as long as possible.

## 4.- Limitations

The main limitation on this study is the lack of a placebo anodal-tDCS group. This means that we will not be able to know whether any benefits that the tDCS group may achieve are related to the theoretical applications that tDCS has, or to the placebo effect. There is also no control over the inclusion of gamification elements, which are added to all interventions that use the GVRE. Another limitation is the lack of a treadmill training and anodal-tDCS group, which would allow us to know to which extent the GVRE adds to the treadmill and the tDCS treatments. However, previous studies have shown no benefits from tDCS combined with treadmill walking on kinematic parameters in PD [64]. For this reason, and also difficulties in achieving a larger sample size, the decision to not include a tDCS + treadmill group was taken. The current study group distribution will allow for clear views on the effects of a customized GVRE to a treadmill set-up, unconditioned by possible placebo effects from tDCS, as well as knowledge on tDCS effects when added to the intervention. Another limitation is also that there is no control on the subtype of PD that any of the participants may be suffering, or whether freezing of gait is present, which could bias the results obtained. Another limitation that should be noted is that currently there is no control over how much physical activity participants follow besides the training program. In some cases, participants feel more engaged to partake on more physical activity, and even though it will be asked to limit physical activity to the usual plus the training protocol, such indications could be ignored.

## Supporting information

S1 Checklist (DOC)

## List of abbreviations

PD: Parkinson’s Disease
PWPD: People with Parkinson’s Disease
tDCS: Transcranial Direct Current Stimulation
GVRE: Gamified Virtual Reality Environment
UKPDSBBC: United Kingdom Parkinson’s Disease Society Brain Bank Criteria
RCT: Randomized Clinical Trial
UPDRS: United Parkinson’s Disease Rating Scale
H&Y: Hoehn & Yahr
PDQ39: Parkinson’s Disease Questionnaire 39
FES-I: Falls Efficacy Scale—International
MoCA: Montreal Cognitive Assessment
MMSE: Mini-Mental State Examination
