# Correction: A treadmill training program in a gamified virtual reality environment combined with transcranial direct current stimulation in Parkinson’s Disease: Study protocol for a randomized controlled trial

**DOI:** 10.1371/journal.pone.0352790

**Published:** 2026-06-29

**Authors:** Pere Bosch-Barceló, Carolina Climent-Sanz, Oriol Martínez-Navarro, Maria Masbernat-Almenara, Anni Pakarinen, Pradip K. Ghosh, Helena Fernández-Lago

The Funding statement for this article is incorrect. The correct Funding statement is as follows: This study has been funded by Instituto de Salud Carlos III (ISCIII – isciii.es) through the project “PI20/00403” (Co-funded by European Regional Development Fund/European Social Fund “A way to make Europe”/”Investing in your future”). ISCIII will be responsible for yearly audition of the trial state.
